# Retraction Note: Long-term follow-up outcomes of MDMA-assisted psychotherapy for treatment of PTSD: a longitudinal pooled analysis of six phase 2 trials

**DOI:** 10.1007/s00213-024-06665-y

**Published:** 2024-08-10

**Authors:** Lisa Jerome, Allison A. Feduccia, Julie B. Wang, Scott Hamilton, Berra Yazar-Klosinski, Amy Emerson, Michael C. Mithoefer, Rick Doblin

**Affiliations:** 1 MAPS Public Benefit Corporations, 1115 Mission St., Santa Cruz, CA 95060 USA; 2 Stanford School of Medicine, Stanford University, Stanford, CA USA; 3 Multidisciplinary Association for Psychedelic Studies, Santa Cruz, CA USA; 4 Medical University of South Carolina, Charleston, SC USA

**Retraction Note: Psychopharmacology (2020) 237:2485–2497**

10.1007/s00213-020-05548-2

The Editors have retracted this article after they were informed of protocol violations amounting to unethical conduct at the MP4 study site by researchers associated with this project. The authors have subsequently confirmed that they were aware of these violations at the time of submission of this article, but did not disclose this information to the journal or remove data generated by this site from their analysis. Additionally, the authors also did not fully declare a potential competing interest. Several of the authors are affiliated with either the Multidisciplinary Association for Psychedelic Studies (MAPS) or MAPS Public Benefit Corporation (MAPS PBC), a subsidiary that is wholly owned by MAPS. As is stated in the Funding declaration, MAPS fully funded and provided the MDMA that was used in this trial, and MAPS PBC organised the trial.

Allison A. Feduccia agrees with this retraction but disagrees with the wording of the retraction notice. Michael C. Mithoefer, Lisa Jerome, Berra Yazar-Klosinski, and Rick Doblin disagree with this retraction. The remaining authors did not respond to correspondence from the Publisher about this retraction.

